# Pesticide Residues Reduce Bacterial Diversity but Enhance Stability via Network Motif Restructuring

**DOI:** 10.3390/toxics13121052

**Published:** 2025-12-04

**Authors:** Chaonan Wang, Ruilin Wu, Xingyan Xue, Cunlu Li, Shengxing Long, Fuliu Xu

**Affiliations:** 1 MOE Laboratory for Earth Surface Processes, College of Urban & Environmental Sciences, Peking University, Beijing 100871, China; wangchaonan@ustc.edu.cn (C.W.);; 2 State Key Laboratory of Advanced Environmental Technology, Department of Environmental Science and Engineering, University of Science and Technology of China, Hefei 230026, China; 3 CCCC Water Transportation Consultants Co., Ltd., Beijing 100007, China

**Keywords:** pesticide residues, motif-based analysis, network stability, null model analysis, resilience modeling, greenhouse cultivation

## Abstract

Agricultural intensification in greenhouse systems leads to a substantial accumulation of pesticides, yet its role in reshaping soil microbial interactions and their network stability remains poorly understood. This study reveals a critical ecological paradox: contrary to classical theory, greenhouse soils under chronic pesticide contamination exhibit significantly enhanced network stability (quantified as the robustness of network global efficiency under targeted node removal simulations) despite a concurrent sharp decline in bacterial diversity. We investigated this counter-intuitive phenomenon by integrating 16S rRNA sequencing, motif-based network analysis, and resilience modeling. Our findings suggest that this enhanced stability is not explained by species richness but, rather, coincides with a fundamental restructuring of the network’s local interaction architecture. Pesticide residues, acting as a strong deterministic selection pressure, shaped the microbial community into a “low-aggregation, high-redundancy” network topology. This was characterized by a decrease in highly clustered, “brittle” interaction motifs (e.g., M3-2) and an increase in sparse triangular anti-motifs (e.g., M3-1). This new architecture mitigates the risk of cascading failures, thereby elevating the network’s collapse threshold. Triazole fungicides (e.g., Tricyclazole and Hexaconazole) were significantly associated with this structural shift. Our study establishes a novel mechanistic link from pesticide stress to motif-level restructuring and enhanced system stability, offering new insights for assessing the health of highly stressed agricultural ecosystems.

## 1. Introduction

In soil ecosystems, the stability of microbial communities provides the dynamic foundation for sustaining soil ecological services, such as participating in nutrient cycling and degrading organic pollutants [1,2,3]. This stability, however, is under severe challenge from global agricultural intensification. Facility cultivation (i.e., greenhouse) systems, as a high-yield agricultural model, create a distinctive soil ecosystem characterized by high cropping indices and frequent agronomic activities [4]. Critically, the long-term application of high-dose, multi-type pesticides to ensure yields results in substantial residue accumulation, far exceeding that of open fields [5,6,7]. This accumulation constitutes an intense, chronic chemical press disturbance, acting as a potent selective filter that significantly reshapes the composition of soil microbial communities [8,9,10,11]. An urgent ecological question therefore arises: how does the stability of the soil microbiome respond to this intense, pesticide-driven selection pressure, and what are the underlying interaction mechanisms?

Complex interactive relationships exist among microbial communities, encompassing competitive dynamics arising from ecological niche overlap and synergistic positive interactions [12,13]. Molecular ecological network (MEN) analysis has become a powerful tool for inferring these interaction patterns [14,15,16]. However, previous studies have often relied on global topological metrics (e.g., connectance, clustering coefficients) for network assessment [17,18,19]. This top-down perspective can obscure a critical fact: ecological networks with similar global structures may exhibit vastly different local interaction patterns [20]. These local patterns, rather than global metrics, may function as the critical “switches” determining whether a community collapses in the face of external disturbances, such as pesticide stress. Therefore, unraveling the mechanisms that sustain microbial network stability requires moving beyond global indices to dissect the micro-scale connectivity patterns that constitute the network.

Network motifs, defined as isomorphic subgraphs that recur in real networks at frequencies far exceeding random chance, represent the fundamental “building blocks” of network interactions [21]. Motif analysis has proven critical for understanding network function and resilience in gene transcription and protein interaction networks [22,23,24]. Applying this bottom-up approach to soil microbial ecology allows for the precise identification of specific local interaction structures that are selected for under pesticide stress. Despite its potential, the application of motif analysis to unravel stability mechanisms in pedogenic microbial communities under chronic chemical stress is nascent. Herein, we systematically compared the bacterial community networks in soils from within and outside greenhouses (i.e., pesticide-contaminated vs. pesticide-free) by integrating 16S rRNA sequencing, motif-based network analysis, and resilience modeling.

We proposed a central hypothesis: (1) The high pesticide load in greenhouse soils acts as a strong deterministic selection filter, which will significantly reduce bacterial diversity and diminish the role of stochastic processes in community assembly. (2) More critically, we posit that this intense selection pressure will actively reshape the interaction architecture of the bacterial network, manifested as a significant reduction in closed triplets and a concurrent increase in open triplets. (3) Ultimately, we hypothesize that it is this pesticide-driven restructuring of local interactions, rather than biodiversity, that serves as the key mechanism driving the enhancement of bacterial network stability in contaminated soils. We expect to identify specific pesticide residues as the key environmental factors driving these structural and functional changes.

## 2. Materials and Methods

### 2.1. Site Descriptions and Sampling

Shouguang (118°32′ to 119°10′ E, 36°41′ to 37°19′ N), located in Shandong province, stands as China’s largest facility vegetable production base, boasting a history of several decades in cultivation. The long-term production of facility vegetables inevitably resulted in an increase in pesticide residues in soil. Therefore, Shouguang’s greenhouses serve as ideal sites for investigating the impact of pesticide residues on the stability of soil microbial networks. In June of 2019, we arranged 64 sampling points across the greenhouse planting areas, comprising 33 greenhouse sites (pesticide-contaminated soils: PC) and 31 open-field sites (pesticide-free soils: PF), respectively (Table S1 and Figure S1). To ensure spatial independence and avoid spatial autocorrelation, the distance between any two sampling sites was kept at greater than 500 m. Regarding the replication strategy, we employed a composite sampling method to ensure representativeness. At each site, surface soil (0–10 cm) was collected from five randomly selected points using a sterile soil auger and thoroughly mixed to form one composite biological replicate. For the open-field samples, soil that had not been treated with pesticides was collected from the area in front of the greenhouse. Each soil sample was split into two parts and transported back to laboratory for further analysis. One portion was stored in a refrigerator at −20 °C for pesticide residue analysis and processed within two weeks to minimize chemical degradation, while the other portion was stored in an ultra-low temperature refrigerator at −80 °C for 16S rRNA sequencing (processed within two weeks) to prevent DNA degradation and preserve community composition.

### 2.2. Physicochemical and Pesticide Residues Analysis

After removing impurities (such as crop roots, etc.), freezing dry, grinding, and passing through a 0.212 mm mesh, soil samples were used for further analysis. Soil pH, the content of total carbon, and organic carbon were analyzed in our laboratory. The residues of seventeen pesticides were extracted with SPE magnesium sulfate Extraction Pouch (MgSO_4_ 6 g, sodium acetate 1.5 g) and then determined by a high-performance liquid chromatography analyzer (Waters Corporation, Milford, MA, USA) [6]. Method validation was performed to ensure accuracy and precision. The average recoveries ranged from 89.32% to 96.15% with relative standard deviations (RSDs) between 2% and 5%. Strict instrument calibration was conducted using external standards (R^2^ > 0.99). Detailed limits of detection (LODs), quantification (LOQs), and extraction procedures are provided in the Supplementary Methods. The detailed analysis of soil properties and pesticide residues can be found in the Supplementary Methods.

### 2.3. DNA Extraction and Amplicon Sequencing

Total genome DNA from soil samples was extracted using the EZNA^®^ Soil DNA Kit (Omega Bio-tek, Norcross, GA, USA) according to manufacturer’s protocol. DNA concentration and purity was monitored on 1% agarose gels. According to the concentration, DNA was diluted to 1 ug/μL using sterile water. The V4-V5 hypervariable regions of bacterial 16S rRNA gene were amplified with the forward primer 515F (5′-GTGCCAGCMGCCGCGG-3′) and the reverse primer 907R (5′-CCGTCAATTCMTTTRAGTTT-3′). The PCR reactions and bacterial high-throughput sequencing and bioinformatics methods were described in detail and followed [8]. PCR amplification was performed as follows: 27 cycles of amplification were performed with an annealing temperature of 55 °C using FastPfu Polymerase. All reactions were conducted in triplicate to ensure reproducibility. Bacterial sequences were rarefied to 21,982 (the minimum reads of samples) sequences per sample. Rarefaction analysis confirmed that this sequencing depth was sufficient to capture the majority of bacterial diversity (Figure S2).

### 2.4. Analyses of Community Diversity and Assembly Mechanisms

PCoA analysis was performed by the Bray–Curtis distance and PERMANOVA analysis was further carried out based on distance matrices to compare the dissimilarity of bacterial communities among different habitats. Community α-diversity was quantified using Shannon and Chao indexes based on the rarefied OTU (operational taxonomic units) table to standardize sequencing depth and ensure comparability across samples. The α and β diversity was calculated using the “microeco” R package (version: 0.17.0) [25]. The average variation degree (AVD) indicated the whole community stability. Mathematically, AVD is defined as the average standard deviation of the relative abundances of all OTUs within a group [26], calculated as an equation (Equation (1)). Ecologically, AVD reflects the magnitude of compositional fluctuation in response to environmental heterogeneity or stochastic drift. Therefore, a lower AVD value indicates a more convergent community structure and higher stability.

(1) $$\mathrm{AVD}=\frac{\sum_{i=1}^{n}\frac{\left|x_{i}-{\bar{x}}_{i}\right|}{\delta_{i}}}{k\;\times \;n}$$

*k*: the number of samples in one sample group.

*n*: the number of *OTU_i_* in each sample group.

*x_i_*: the abundance of *OTU_i_* in each sample.

${\bar{x}}_{i}$: the mean of the abundance of *OTU_i_* across all samples.

$\delta_{i}$: the standard deviation of the abundance of *OTU_i_* across all samples.

Three methods were performed to measure the potential importance of stochasticity in metacommunity assembly using “iCAMP” R package (version: 1.5.12) [27]. We calculated the stochastic ratio (Normalized Stochasticity ratio, NST) to consider the relative contributions of stochasticity [28]. The five community assembly mechanisms were relative quantified based on βNTI (beta Nearest Taxon Index) and RCbray (Bray–Curtis-based Raup Crick metrics) [29]. To quantify the relative importance of ecological processes, the iCAMP analysis (v1.5.12) was performed [27]. First, a phylogenetic tree was constructed using FastTree based on the aligned representative sequences (https://morgannprice.github.io/fasttree/, accessed on 15 October 2023). Phylogenetic binning was then conducted to divide OTUs into different groups with a phylogenetic signal threshold (*d_s_*) of 0.2 and a minimum bin size (*n_min_*) of 24. Within each bin, a null model analysis was performed using 1000 randomizations (taxa shuffle) to calculate *βNTI* and *RC_bray_*. Based on these metrics, the community assembly was partitioned into homogeneous selection, heterogeneous selection, dispersal limitation, homogenizing dispersal, and undominated processes (drift).

### 2.5. Network Construction and Analysis

OTUs with an average abundance < 0.01% and detected in less than half of the corresponding habitat samples were removed, retaining 547 OTUs within the greenhouse and 716 OTUs for the open field for further network computation. Despite containing fewer nodes, the PC network exhibited a higher number of edges (2602) compared to the PF network (2489), confirming that the filtering preserved the core, highly connected microbial structure. The bacterial interaction networks were established using the “psych” (version: 2.2.9) R (version: 4.0.1) package based on Spearman correlation coefficients. We kept strong and significant connections (|*R*| > 0.7 and *p* < 0.05) in bacterial networks to focus on robust biological interactions and minimize false positives derived from environmental noise [30,31].

For the subsequent calculation of topological properties and motif analysis, the networks were treated as binary and undirected graphs, where edges represented valid co-occurrence associations regardless of correlation strength. Based on the zero-order null model (maintaining the same number of nodes and edges for the corresponding real network), 1000 random networks were constructed to compare the topological properties of real networks and corresponding random networks. Subsequently, the average path length (APL) and average clustering coefficient (ACC) were computed for both real and random networks. Additionally, the *Z* statistic and its *p*-value were calculated to quantitatively compare the global topological properties of real and corresponding random networks. Overall topological structures of the microbial co-occurrence networks were calculated in “igraph” (version: 1.3.5) R package. The small-world coefficient (SWC) was calculated to quantitatively assess the network topology. This metric assumes that a small-world network exhibits a clustering coefficient (C) significantly higher than that of random networks (*γ* = C_real_/C_rand_ > 1), while maintaining a comparable average path length (L) (*λ* = L_real_/L_rand_ ≈ 1). Consequently, the coefficient is defined as *σ* = *γ*/*λ*. A network is considered to possess ‘small-world’ properties if *σ* > 1. The random values (C_rand_ and L_rand_) were derived from the average of 1000 Erdős–Rényi random networks with the same number of nodes and edges as the empirical network [32].

### 2.6. Motif Analysis

In order to further explore the differences in the interactions between bacterial networks, we conducted microstructure analysis. The concept of network motif originated from biology and was proposed by Shen-Orr et al. [20]. Generally, motifs represent recurring patterns of interactions within real networks; their occurrence is notably higher compared to random networks possessing an equivalent number of nodes and edges.

In this study, we specifically focused on 3-node and 4-node motifs for two primary reasons. First, Computational Tractability and Interpretability: The number of possible isomorphic subgraph types increases exponentially with motif size (Figure S5). Focusing on smaller motifs avoids the ambiguity associated with interpreting high-order structures. Second, Fundamental Building Blocks: 3- and 4-node motifs represent the fundamental topological primitives that constitute larger, more complex interaction modules. Understanding these basic units is a prerequisite for deciphering global network architecture [24]. Specifically, the configuration and potential ecological significance are illustrated in Table S2 for two distinct types of 3-node subgraphs and six distinct types of 4-node subgraphs.

We identified motifs in bacterial interaction networks through a g-tries algorithm using the analysis tool gtrieScanner [33]. In brief, we constructed 1000 random networks based on first-order null model and then identified subgraphs in both real and random networks. The relevant statistical characteristics used to evaluate the motifs were listed below [21]:

(1) Value *Z* of motif

For a given subgraph *M_i_*, the value *Z* of subgraph *M_i_* in the real network is


(2) $$Z=(N_{\mathrm{reali}}-<N_{\mathrm{randi}}>)/\delta_{\mathrm{randi}}$$

*N_reali_*: the number of times it appears in the real network.

*N_randi_*: the number of times it appears in the random network.

<*N_randi_*>: the mean of *Nrand_i_*.

*δ_randi_*: the standard deviation of *Nrand_i_*.

In case *Z* > 0, the subgraph is a motif. Otherwise, the subgraph is an anti-motif. Generally, a higher value of *Z* indicates greater importance of the motif in the network.

(2) Value *p* of motif

Value *p* refers to the probability that the frequency of subgraph *M_i_* in the random network is greater than in the real network. In this study, we adopted a strict significance threshold of *p* < 0.01. Thus, a subgraph was defined as a network motif only if its Z-score was positive and its occurrence was significantly higher than in randomized networks *p* < 0.01.

### 2.7. Evaluating the System Resilience of Bacterial Network

Due to the diversity and complexity of system responses to disturbances, current studies have not yet found a unified theory to describe the network resilience [34,35]. In this study, the resilience of a network was defined as the maximum degree of disturbance that the network could withstand without collapsing or transition to another stable state [36]. We employed three distinct strategies to simulate extinction: (1) random attacks: randomly selecting and removing a certain proportion of nodes (simulating 1000 replicates); (2) degree-based attacks: removing nodes, in descending order, based on their degree centrality; and (3) betweenness-based attacks: removing nodes, in descending order, based on their betweenness centrality. By simulating species disappearance using these three methods, we assessed the robustness of the network through different types of disruptions and determined whether greenhouse cultivation had an impact on the stability of bacterial communities in soil. The resilience of a network was calculated as follows:

#### 2.7.1. Description of Resilience Measurement Models for Networks

We used the relative size of the global efficiency in networks as a measure of overall network robustness. The ratio *I*(*p*) of the global efficiency *Ep* of the network after removing the “node” ratio *p* to the global efficiency *Epre* of the original network represents the change in bacterial global network efficiency after certain attacks. The closer it is to 1, the stronger the network’s ability to maintain the original network efficiency:

(3) $$I\left(p\right)=\frac{E_{p}}{E_{\mathrm{pre}}}$$

(4) $$E_{\mathrm{pre}}=\frac{2}{N\left(N-1\right)}\sum_{i\neq j}\frac{1}{d_{\mathrm{ij}}^{\mathrm{pre}}}$$

(5) $$E_{p}=\frac{1}{N(N-1)}\sum_{i\neq j}\frac{1}{d_{\mathrm{ij}}^{p}}$$

where *N* represents the number of total vertices, *i* and *j* represent paired nodes, *p* is the proportion of nodes removed, $d_{ij}^{pre}and\;d_{ij}^{p}$ indicates the distances between nodes in the original or removed *p* ratio “node” in networks.

**Figure 1:**
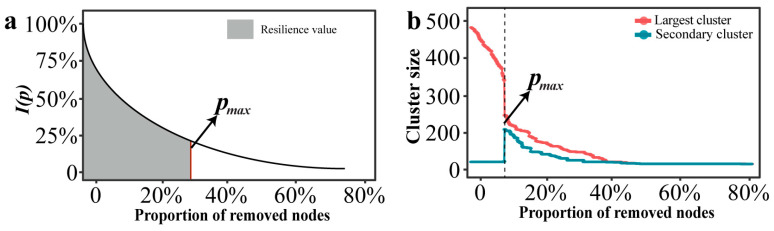
Curve and threshold schematic diagram of network under certain attack. (**a**) Changes in resilience values within a network under attack. (**b**) The threshold of a network under attack.

The resilience of a network is not only reflected in its robustness, but also in maintaining a high level of network efficiency as high as possible before the networks crashes. In this study, we used the network characteristic curve of the global efficiency change rate *I*(*p*) as a function and the maximum allowable failure “node” proportion *p_max_* as a threshold value to characterize the resilience level of a network (Figure 1a). Fitting models were “*y* = *a*·*exp*(*b*·*x*) + *c*” in “ggtrendline” (version: 1.0.3) R package. This non-linear decay trajectory is characteristic of complex networks under disintegration, where connectivity often collapses rapidly after a critical threshold of node removal, a pattern widely observed in network robustness studies [36,37,38]. The resilience index *R* was defined as follows:

(6) $$R\;=\int_{0}^{p_{\max}}I\left(p\right)\mathrm{df}\;=\int_{0}^{p_{\max}}(E_{p}/E_{\mathrm{pre}})\mathrm{df}$$

#### 2.7.2. Identifying the Threshold of a Given Network

In the research of complex networks, the size of the clusters can characterize the connectivity of the network structure [39]. The clusters can be divided into the largest cluster and the secondary cluster. When the nodes failure reaches a certain critical value, the scale of the secondary cluster suddenly increases, while the scale of the largest cluster suddenly decreases. The network collapse threshold (denoted as *p_max_*) was operationally defined based on percolation theory. Specifically, *p_max_* corresponds to the critical fraction of removed nodes where the size of the second-largest connected component reaches its maximum value. This peak marks the critical phase transition point where the giant connected component disintegrates into smaller isolated fragments, signifying network collapse [40,41].

#### 2.7.3. Calculating the Resilience Values

We proposed a metric method based on motifs to evaluate the resilience of local higher-order structures in a network, which represented the local robustness characteristics of bacterial interactions in a network. We analyzed the dynamics of network motifs under three attack strategies and calculated the resilience values of the motifs: (1) as the nodes disappeared, the remaining motif concentration was calculated to observe the stability of the network interaction relationship; (2) fitting the equation of motifs under different attacks; (3) calculating resilience values of different motifs as described by Section 2.7.1 and Section 2.7.2. It should be noted that since the networks were constructed as aggregate entities for each group (metacommunity), biological replicates for network topology were not available. Therefore, the comparative analysis of resilience relies on the distinct trajectories of the robustness curves rather than hypothesis testing based on group variance. Additionally, the R scripts used for network construction and attack simulations have been uploaded to https://github.com/wangyuelang/The-method-of-network-interactions-stability-based-on-motif-analysis-under-different-attacks-in-R (accessed on 28 November 2025).

### 2.8. Linking Abiotic Factors to the Resilience and Interaction of Bacterial Network

Variance partitioning analysis (VPA) was conducted to quantify the relative contributions of soil chemical properties and pesticide residues to the bacterial community within the greenhouse using the “vegan” (version: 2.5-7) R package. We utilized Generalized Linear Mixed Models (GLMMs) to evaluate the effects of environmental predictors. The models were fitted using a Gaussian error distribution with an identity link function, which was appropriate for the continuous nature of our response variables. To account for potential spatial heterogeneity among sampling locations, ‘Sampling Site’ was included as a random effect. The variance was then decomposed using the glmm.hp function in the “glmm.hp“ (version: 1.0-0) R package [42]. The ratio of variance components indicated the relative effect of each environment variable. In addition, we calculated the correlation between each chemical property or pesticide with whole and network OTUs using the “psych” (version: 2.2.9) R package based on Spearman correlation coefficients. All *p*-values were adjusted using the Benjamini–Hochberg (BH) procedure. Only correlations with an adjusted *p*-value (FDR) < 0.05 were considered statistically significant. To gain insights into the functional potential of the bacterial communities, we predicted the functional profiles based on the 16S rRNA gene sequences using the “Tax4Fun“ (version: 1.0) R package [43]. Statistical differences in the relative abundance of functional categories between pesticide-contaminated (PC) and pesticide-free (PF) soils were evaluated using Welch’s *t*-test, with a significance threshold of *p* < 0.05.

## 3. Results

### 3.1. Bacterial Community Diversity, Stability and Ecological Assembly Processes

**Figure 2:**
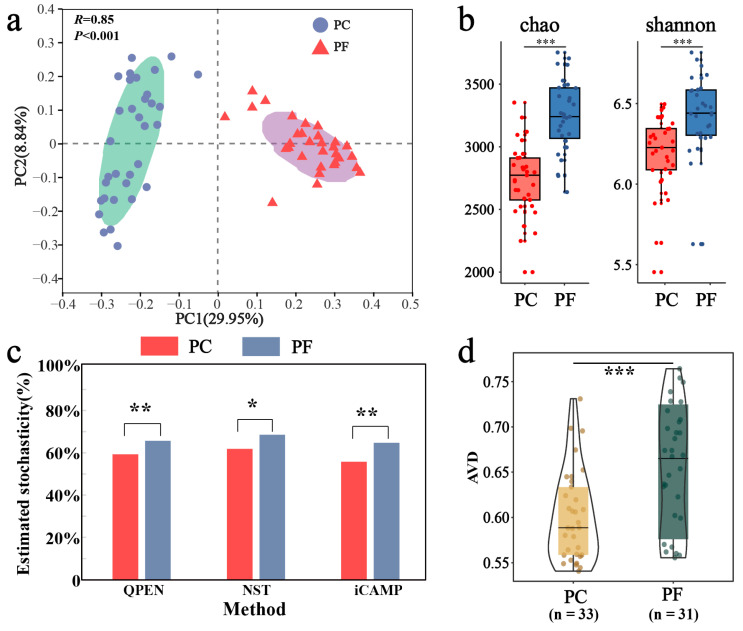
Differences in bacterial community structure and assembly between soils containing pesticide residues and uncontaminated soils. (**a**) Bacterial community beta-diversity visualized using PCoA ordination based on the Bray–Curtis distance. The BETADISPER analysis revealed no significant difference in multivariate dispersion between groups (*p* > 0.05), indicating homogeneity of group dispersions. (**b**) Comparison of alpha-diversity by Chao and Shannon indices. (**c**) Comparison of stochastic processes inside and outside greenhouses by three assembly models (QPEN, NST, iCAMP). (**d**) Comparison of community stability from greenhouse to open field using average variation degree (AVD) indices. *: *p* < 0.05; **: *p* < 0.01; ***: *p* < 0.001.

Our PCoA and PERMANOVA tests revealed distinct separation in bacterial community composition between pesticide-contaminated soils and pesticide-free soils (R = 0.85, *p* < 0.001; Figure 2a). Additionally, the richness and diversity of pesticide-contaminated soils were significantly lower than those of pesticide-free soils (Figure 2b). To investigate the driving factors behind bacterial community differences between these soil types, ecological processes were analyzed. Results from three community assembly models indicated that stochastic processes dominated bacterial communities in both soil types (>50%), with significantly higher stochasticity observed in pesticide-free soils compared to pesticide-contaminated soils (Figure 2c). The iCAMP model further quantified these processes, with drift and others (34.8%) and homogeneous selection (31.6%) being the main processes for bacterial communities in pesticide-free soils, while homogeneous selection (43.9%) dominated in pesticide-contaminated soils (Figure S3). Furthermore, the AVD results provided quantitative insights into community stability. A lower AVD value indicates that the community composition is more convergent and resistant to stochastic fluctuation (i.e., higher stability), likely due to strong deterministic environmental filtering. Conversely, a higher AVD suggests a more divergent and dynamic community structure. In this study, the significantly lower AVD in pesticide-contaminated soils compared to pesticide-free soils indicates that pesticide residues acted as a strong selective force, constraining community variation and enhancing structural stability (Figure 2d).

### 3.2. Differences in Topology Properties and Motif Characteristics

We constructed bacterial networks comprising two types of habitats: pesticide-contaminated and pesticide-free soils (Figure S4). The APL and ACC in all real networks were significantly higher than those in random networks (*p* < 0.01) based on the zero-order null model (1000 random networks) (Table 1). This indicated the prevalent presence of non-random interactions in the real networks. Furthermore, the bacterial network in pesticide-contaminated soils exhibited a significantly more complex topology than in pesticide-free soils. This complexity was quantitatively confirmed by a higher edge density (PC: 0.017 vs. PF: 0.01) and modularity (PC: 0.045 vs. PF: 0.063) (Table 1). Despite having fewer nodes (547 vs. 716), the PC network possessed more edges (2602 vs. 2489) and a higher proportion of negative correlations, indicating a denser and more interconnected interaction structure under chemical stress. All real networks displayed small-world characteristics, as evidenced by their small-world coefficients (SWC > 1).

In order to explore the microstructural characteristics of bacterial interaction networks, we systematically examined the 3–8-node subgraphs of each bacterial network (Figure S5). The results indicated that the types and absolute quantities of subgraphs in the bacterial networks exponentially increased with the size of the subgraphs under investigation. For instance, within the network of greenhouse soil, there were 6 types and 1.67 × 10^6^ individuals of 4-node subgraphs, whereas there were 11,088 types and 3.72 × 10^11^ members of 8-node subgraphs. Hence, for distinct presentation and convenient analysis, this study focused on comparing 3-node and 4-node subgraphs.

For the 3-node subgraphs, the subgraph M3-2 consisted of three interconnected nodes, representing motifs (*Z* > 0) within the network. Conversely, the subgraph M3-1 was characterized by a structure where one node was connected to two nodes that were not connected to each other, representing anti-motifs (*Z* < 0) within the networks. The bacterial network of pesticide-free soils exhibited a higher concentration of M3-2 (20.97%:16.36%) compared to pesticide-contaminated soils, indicating a higher clustering feature, which aligned with the ACC results of overall topological property (Table 1 and Table 2). As for the 4-node subgraphs, the subgraphs M4-1, M4-2, and M4-3 had relatively higher concentrations in all networks, serving as the main microstructural forms of bacterial network interactions (Table 2). The subgraphs M4-3, M4-5, and M4-6 exhibited motif patterns (*Z* > 0) in the bacterial networks, appearing much more frequently in the real networks compared to random networks. These subgraphs all contained a stable triangular structure involving pairwise interactions (M3-2), possibly suggesting a general interaction rule that governed the behavior of bacterial networks. Furthermore, M4-5 and M4-6 exhibited significantly higher relative concentrations in the pesticide-free networks compared to the pesticide-contaminated networks, suggesting a greater density of localized interactions in the bacterial network of pesticide-free soils. To quantify the extent of motif enrichment beyond random chance, we calculated the effect size ratio (C_real_/C_rand_). Notably, while complex clique-like structures were identified as motifs in both networks, their enrichment intensity differed drastically. The effect size ratio for M4-6 in the pesticide-free network (Ratio = 84.45) was approximately 8-fold higher than that in the pesticide-contaminated network (Ratio = 10.55) (Table 2). This sharp decline suggests that although these cooperative modules persist under stress, their formation is strongly suppressed by pesticide residues.

### 3.3. Resilience Analysis in Bacterial Networks Under Attacks

**Figure 3:**
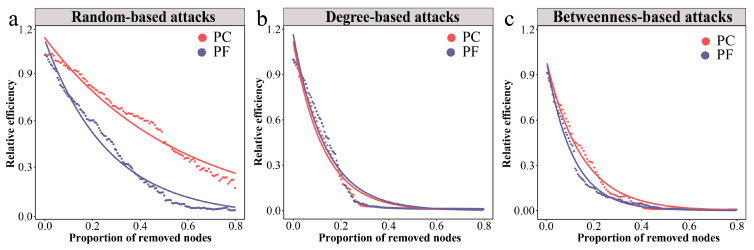
Dynamics of relative global efficiency in bacterial networks under different attacks. (**a**) Changes in relative global efficiency under random-based attacks. (**b**) Changes in relative global efficiency under degree-based attacks. (**c**) Changes in relative global efficiency under betweenness-based attacks. Larger shifts upon the same proportion indicate that there is less stability within subgraph types.

We employed the network’s relative global efficiency as an indicator to assess the overall robustness of bacterial networks under three different attack methods. The results demonstrated that the relative global efficiency of the pesticide-contaminated soil network declined at a slower rate under random attacks compared to the pesticide-free soil network, displaying higher robustness (Figure 3a). When subjected to intentional attacks, both the pesticide-contaminated and pesticide-free soil networks exhibited similar reductions in relative global efficiency, showing lower robustness than when under random attacks (Figure 3b,c). To further quantify the resilience of bacterial networks, we first calculated the threshold values of network collapse (Figure S6). Subsequently, we employed a resilience measurement model to calculate the resilience values under the three attack scenarios. The findings revealed that the pesticide-contaminated soil network had higher resilience than the pesticide-free soil network under all attack types, underscoring the greater stability of bacterial communities in pesticide-contaminated soils (Table 3).

**Figure 4:**
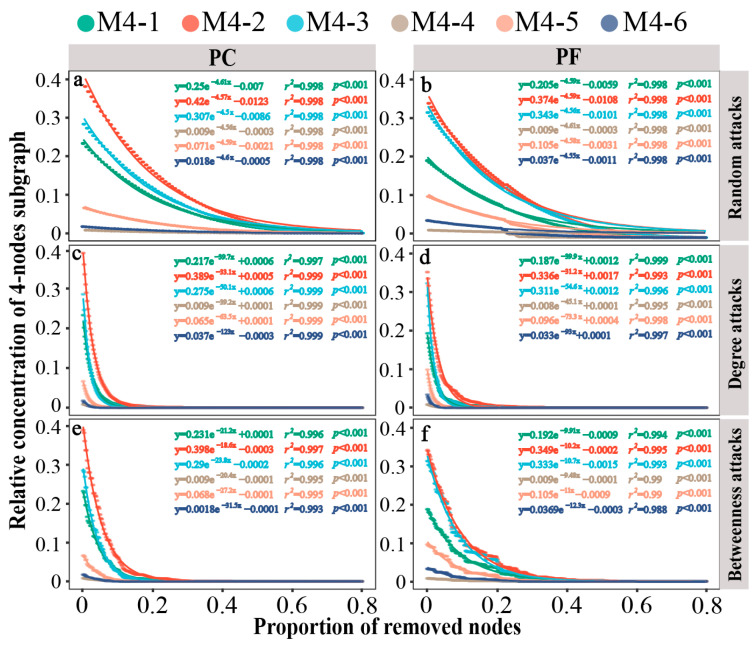
Dynamics of relative concentrations of different 4-node subgraphs in bacterial networks under different attacks. (**a**) network of greenhouse under random-based attacks; (**b**) network of open field under random-based attacks; (**c**) network of greenhouse under degree-based attacks; (**d**) network of open field under degree-based attacks; (**e**) network of greenhouse under betweenness-based attacks; (**f**) network of open field under betweenness-based attacks. Larger shifts upon the same proportion indicate that there is less stability within subgraph types. PC: pesticide-contaminated; PF: pesticide-free soils.

We further evaluated the microstructural resilience of network interactions. In pesticide-contaminated and pesticide-free soil bacterial networks, the 3-node motifs and anti-motifs exhibited similar rates of decline under random attacks, despite having different starting concentrations (−3.34 or −3.35; Figure S7a,b). Similarly, the 4-node subgraphs showed consistent results (Figure 4a,b). In the face of intentional attacks, the motif (M3-2) disappeared at a faster rate compared to the anti-motif (M3-1) in both bacterial networks, particularly in degree-based attacks (Figure S7c–f). For the 4-node subgraphs, motif M4-6 exhibited the fastest rate of disappearance under all intentional attacks, followed by the motifs M4-5 and M4-3, indicating lower robustness of motifs compared to anti-motifs under intentional attacks (Figure 4c–f). Furthermore, quantitative comparison of the resilience values of subgraphs in the bacterial networks of pesticide-contaminated and pesticide-free soils were conducted (Table S3). Results showed that motifs in pesticide-contaminated soils had lower resilience levels than those in pesticide-free soils across all three attack types, regardless of motif size (3-node or 4-node).

### 3.4. Influence of Chemical Properties and Pesticide Residues on Bacterial Communities and Networks

**Figure 5:**
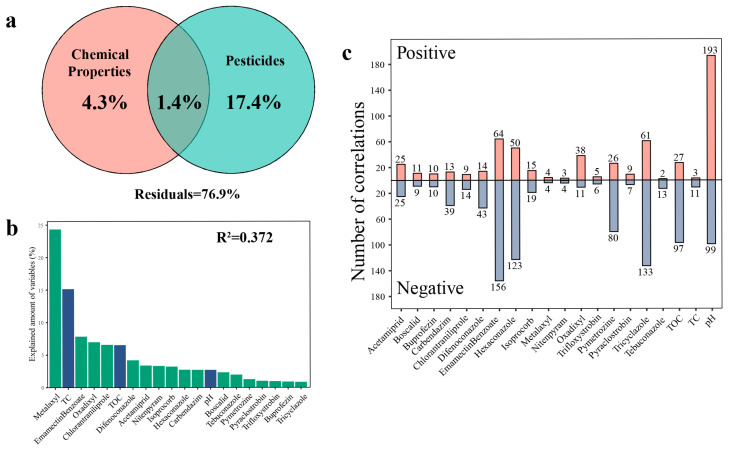
Impact of abiotic factors on the abundance of individual OTUs within pesticide-contaminated soil. (**a**) Variance partition analysis showing relative contributions of chemical properties and pesticide residues to the community variations based on Bray–Curtis distance. (**b**) Variance partitioning of different environmental variables at AVD values. Green bars: pesticide types; Blue bars: physicochemical properties. (**c**) Number of OTUs that significantly changed their abundance in response to abiotic factors of network members. The number at the top of the bar represents the number of OTUs significantly related to corresponding environmental variable. TC: total carbon; TOC: total organic carbon.

Chemical properties and pesticide residues are listed in Table S4. Variance partition analysis showed that the abiotic factors could explain 23.1% of community variations, of which 17.4% could be explained separately by pesticide residues (Figure 5a). Subsequently, we examined the correlation between the chemical properties and pesticide residues within the greenhouse’s soil and the relative abundance of 6010 OTUs (Figure S8). The results indicated that pH was significantly correlated with the largest number of OTUs (1220), followed by Emamectin Benzoate (861), Tricyclazole (797), and Hexaconazole (707). Based on the mixed linear model and variance decomposition results, it was determined that Metalaxyl among all environmental variables contributed more to the total variation (24.32%), followed by TC (15.13%) and Emamectin Benzoate (7.84%) (Figure 5b). These results indicated that the stability of soil bacterial communities was influenced by both pesticide residues and physicochemical factors. Furthermore, we analyzed the correlation between 547 OTUs that comprised the PC network (accounting for 9.1% of the entire bacterial community) and the abiotic factors (Figure 5c). Our results indicated that different types of environmental variables were significantly correlated with 7 to 220 different OTU individuals, respectively. Among these, pH exhibited a significant correlation with the largest number of OTUs (292), followed by Emamectin Benzoate (220), Tricyclazole (194), and Hexaconazole (173). Our results demonstrated that OTUs sensitive to pesticide residues were widely distributed throughout the entire bacterial network, indicating a substantial impact of pesticide residues on the bacterial network. The Tax4Fun prediction revealed distinct functional profiles between the two groups (Figure S9). Consistent with the chemical stress environment, the PC soils exhibited a significantly higher abundance of genes associated with ‘Xenobiotics biodegradation and metabolism’ (*p* < 0.001), indicating a functional selection for pesticide-degrading traits. Furthermore, pathways related to basic survival and maintenance, including ‘Carbohydrate metabolism’ (*p* < 0.01), ‘Amino acid metabolism’ (*p* < 0.001), and ‘Energy metabolism’ (*p* < 0.001), were significantly enriched in PC soils. In contrast, functions associated with biotic interactions and environmental sensing were significantly suppressed in the contaminated soils. Specifically, ‘Signal transduction’ (*p* < 0.001), ‘Cell motility’ (*p* < 0.001), and ‘Membrane transport’ (except for specific efflux pumps) showed lower abundances in PC compared to PF soils.

## 4. Discussion

The central finding of this study reveals a phenomenon that challenges the classical “diversity-begets-stability” hypothesis: compared to open fields, greenhouse soils under chronic pesticide contamination exhibit significantly enhanced community network stability (resilience) despite a concurrent loss of bacterial diversity. This finding directly addresses the core question posed in the Introduction regarding how chronic chemical stress (pesticide residues) reshapes soil microbial stability. Our results demonstrate that under intense external selection pressure, community stability is not dictated by species richness but, rather, by the architectural restructuring of its interaction network.

Our first hypothesis was validated. The assembly of bacterial communities in greenhouse soils was dominated by deterministic processes (particularly homogeneous selection), with the role of stochastic processes significantly diminished (Figure S3). This aligns with the observed reduction in alpha-diversity (Figure 2b). Previous studies have indicated that single and continuous cultivation approaches substantially reduced microbial activity and biomass in the soil [44,45], which could explain the decline in bacterial diversity observed in greenhouse soil. The high-concentration, multi-type pesticide residues (Figure 5a) constitute a strong, persistent environmental filter, permitting only microorganisms capable of tolerating or degrading these compounds to thrive [46,47,48]. This intense, pesticide-driven deterministic selection leads to the “biotic homogenization” of the community [49,50], setting the compositional stage for the subsequent fundamental shifts in network architecture.

The key mechanistic insight of this study, confirming our second and third hypotheses, lies in the network’s structural adaptation. Firstly, through zero-order null models, there were significant distinctions between the actual network topology and the corresponding random network, demonstrating that the networks were non-randomly constructed (Table 1). The overall network topology indicated that the frequency of interactions within the greenhouse soil was significantly higher than that observed in the bacteria network of open field (Table 1 and Figure S4). Because of the continuous input of fertilizers and root secretions, the greenhouse soil is characterized by a nutrient-enriched environment. Our physicochemical analysis confirmed this pattern: the contents of total organic carbon (TOC) in greenhouse soils were 14.3 g/kg, respectively, which were significantly higher than those in open-field soils (Table S4) [51]. The elevated nutrient availability provides stable energy resources [52,53], allowing bacteria to strongly modify the chemical environment and fostering a greater number of interactions [54]. Consequently, the bacterial network within the greenhouse soil exhibited fewer nodes while producing a higher number of edges. Secondly, our results show that while the greenhouse network possesses more total connections, its local interaction clustering is significantly lower than in the open field (Table 2). This indicates that the pesticide-stressed network evolved a “low-aggregation, high-redundancy” topology. Crucially, this trend is even more pronounced in higher-order structures. The sharp decline in highly connected 4-node motifs (e.g., M4-5 and M4-6) in contaminated soils offers a mechanistic explanation for the observed stability.

Ecologically, these clique-like motifs represent ‘tightly coupled functional guilds’ or obligate cross-feeding relationships, which require high metabolic costs to maintain multiple interspecific connections [55,56,57]. Under chronic pesticide stress, maintaining such complex cooperation becomes energetically unfavorable. In the open field, these highly clustered motifs (M3-2, M4-6) likely represent efficient cooperative units. However, these rigid structures are prone to ‘co-extinction cascades’—the pesticide-induced removal of one key node can destabilize the entire module. Conversely, the prevalence of sparse motifs (e.g., M3-1) and the reduction in brittle cliques in the greenhouse network, while potentially sacrificing local cooperative efficiency, significantly enhances overall redundancy. In such an architecture, the removal (extinction) of a single node is less likely to trigger a systemic chain reaction. This aligns with theoretical models demonstrating that excessive positive connectivity in microbial networks can amplify perturbations, leading to instability [58]. Conversely, structural sparsity and the reduction in unstable feedback loops (often embedded in complex cliques) serve to dampen the propagation of disturbances [59], thus manifesting as higher macroscopic resilience (Table 3).

Further analysis identified the key environmental factors associated with this network restructuring (Figure 5c). Consistent with previous studies [60,61], soil pH emerged as a significant factor. Mechanistically, soil pH acts as a fundamental physiological filter, imposing strong deterministic selection on microbial community assembly. Since most bacterial taxa exhibit narrow pH optima for growth, shifts in pH can directly exclude maladapted species, thereby altering the pool of potential network nodes [60,62]. Concurrently, multiple pesticide residues—particularly triazole fungicides (e.g., Tricyclazole, Hexaconazole)—were significantly correlated with the abundance of numerous network OTUs (over 24% of total network nodes). Notably, variance partitioning analysis highlighted that metalaxyl explained the largest proportion of community variation (24.32%) among all environmental variables (Figure 5b). This dominant influence can be attributed to its unique physicochemical properties. As a systemic fungicide with high water solubility and a low soil adsorption coefficient (*K_oc_*), metalaxyl exhibits exceptional mobility and bioavailability in soil pore water [63]. This allows it to exert a widespread and persistent selective pressure on the soil microbiome, potentially disrupting fungal–bacterial interactions and reshaping the community structure more profoundly than less mobile hydrophobic pesticides [64]. These findings support our hypothesis that these specific chemicals are the primary source of the selection pressure. By potentially inhibiting sensitive species (causing diversity loss) and removing specific nodes, they appear to have indirectly and systematically contributed to the selection of the aforementioned “low-aggregation, high-redundancy” network interaction pattern, ultimately coinciding with enhanced stability. Additionally, the potential for certain microorganisms to utilize pesticides as a carbon source may further shape these interactions [65,66].

Our functional prediction analysis provides strong mechanistic evidence linking pesticide stress to network restructuring. The specific enrichment of ‘Xenobiotics biodegradation and metabolism’ in greenhouse soils (Figure S9) corroborates that pesticide residues act as a primary selective force, favoring taxa capable of metabolizing these chemical stressors as energy sources. Crucially, the functional shifts offer a biological explanation for the observed simplification of network motifs. We observed a significant downregulation of ‘Signal transduction’ and ‘Cell motility’ pathways in contaminated soils. This ‘functional uncoupling’ aligns perfectly with the ‘low-aggregation’ topological shift we observed, reinforcing the trade-off between survival stability and complex biotic interactions [67]. Furthermore, it is crucial to recognize that this enhanced network stability may come at an ecological cost. While the restructured network is more resilient to collapse under pesticide stress, this ‘survival-mode’ topology might be less efficient in performing broad ecosystem functions, such as nutrient cycling or organic matter turnover, compared to the diverse and highly clustered networks found in pristine soils. Thus, the observed resilience represents a fundamental trade-off: the community prioritizes survival stability over functional diversity.

Overall, as a manifestation of intensive agricultural activities, pesticide residues were widely associated with OTUs throughout the bacterial network within the greenhouse, suggesting a strong link to the observed shifts in interspecific interactions and network stability. However, it is important to acknowledge the methodological limitations inherent in molecular ecological network analysis. The co-occurrence patterns inferred here represent statistical associations, which do not necessarily equate to direct biological interactions (e.g., competition or symbiosis). These associations may also arise from shared habitat preferences or common responses to environmental drivers [68,69]. Therefore, while our motif-based approach provides robust structural insights, future experimental validations (e.g., co-culture assays) are required to definitively verify the specific interactions hypothesized in this study.

## 5. Conclusions

In conclusion, this study provides novel mechanistic insights into the diversity–stability relationship in soil microbiomes under the context of intensive agriculture. Our results suggest that pesticide stress, acting as a strong deterministic filter, is associated with reduced community diversity while coinciding with a restructuring of the network interaction architecture at the motif level. This structural shift favors a more resilient topology, which may explain the observed enhancement in system stability (counter-intuitively). These findings highlight that when assessing the health of stressed ecosystems, moving beyond traditional diversity metrics to analyze the structural properties (especially motifs) of the interaction network may be critical. Future sustainable agricultural management should focus not only on ‘conserving biodiversity’ but also on ‘maintaining a resilient network interaction structure’.

## Figures and Tables

**Table 1:** Key topological features of bacterial networks.

Group	Nodes	Edges	AD ^1^	APL ^2^			ACC ^3^			SWC ^4^	Proportion of Negative Edges	ED ^5^	Md ^6^
				L_real_	L_null_	*Z*	C_real_	C_null_	*Z*				
PC	547	2602	9.51	2.83	2.54 ± 0.06	−977.34 **^7^	0.37	0.16 ± 0.01	−894.89 **^7^	5.1	28.56%	0.017	0.045
PF	716	2489	6.95	4.01	2.45 ± 0.05	−1002.00 **^7^	0.44	0.17 ± 0.01	−945.19 **^7^	4.29	8.48%	0.01	0.063

^1^ AD: average degree. ^2^ APL: average path length. ^3^ ACC: average clustering coefficient. ^4^ SWC: small-world coefficient. ^5^ ED: Edge Density. ^6^ Md: Modularity. ^7^ **: *p* < 0.01.

**Table 2:** Subgraph’s features of bacterial networks.

ID	Subgraph Shape	Group	C_real_ ^1^	C% ^2^	C_rand_ ^3^ ± C_std_ ^4^	*Z* ^5^	Ratio	Motif Type
M3-1	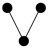	PC	49,321	83.64%	66,636.99 ± 289.61	−59.79	0.74	Anti-motif
		PF	29,372	79.03%	48,461.97 ± 162.38	−117.57	0.61	Anti-motif
M3-2	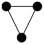	PC	9649	16.36%	3877.01 ± 96.54	59.79	2.49	Motif
		PF	7794	20.97%	1430.68 ± 54.13	117.57	5.45	Motif
M4-1	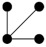	PC	388,142	23.30%	795,491.54 ± 10,492.40	−38.82	0.49	Anti-motif
		PF	136,074	19.15%	47,5103.86 ± 5054.21	−67.08	0.29	Anti-motif
M4-2	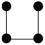	PC	649,095	38.96%	1,149,441.21 ± 7469.36	−66.99	0.56	Anti-motif
		PF	247,593	34.85%	747,524.74 ± 4081.13	−122.5	0.33	Anti-motif
M4-3	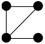	PC	475,752	28.56%	320,565.61 ± 6095.17	25.46	1.48	Motif
		PF	227,266	31.99%	108,099.04 ± 3792.96	31.42	2.10	Motif
M4-4	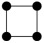	PC	13,622	0.82%	36,048.90 ± 791.71	−28.33	0.38	Anti-motif
		PF	5879	0.83%	13,552.88 ± 427.80	−17.94	0.43	Anti-motif
M4-5	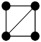	PC	110,744	6.65%	36,566.47 ± 1692.55	43.83	3.03	Motif
		PF	69,747	9.82%	6996.18 ± 555.03	113.06	9.97	Motif
M4-6	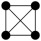	PC	28,670	1.72%	2717.98 ± 283.25	91.62	10.55	Motif
		PF	23,873	3.36%	282.69 ± 49.66	475	84.45	Motif

^1^ C_real_: the number of the subgraphs appearing in the real network. ^2^ C%: the frequency of the subgraphs appearing in the real network. ^3^ C_rand_: the average frequency of the subgraphs appearing in the random network. ^4^ C_std_: the standard deviation of the subgraphs appearing in the random network. ^5^ *Z*: *Z* values of the motifs.

**Table 3:** Resilience values and fitting formula of global efficiency in bacterial networks under different attacks.

Attacked Types	Group	Fitting Formula	*R* ^2^	*p*-Values	Threshold	Resilience
Random	PC	y = 5.3 e^−0.224x^ − 4.26	0.994	*p* < 0.001	0.8044	0.4745
	PF	y = 1.2 e^−2.65x^ − 0.158	0.99	*p* < 0.001	0.5293	0.2578
Degree	PC	y = 0.243 e^−7.85x^ − 0.0018	0.979	*p* < 0.001	0.2925	0.0273
	PF	y = 0.18 e^−7.48x^ − 0.0016	0.969	*p* < 0.001	0.2542	0.0201
Betweenness	PC	y = 0.227 e^−6.55x^ − 0.003	0.989	*p* < 0.001	0.4059	0.031
	PF	y = 0.159 e^−9.33x^ − 0.0011	0.99	*p* < 0.001	0.1215	0.0117

## Data Availability

The original contributions presented in this study are included in the article/Supplementary Materials. Further inquiries can be directed to the corresponding author.

## Supplementary Materials

The following supporting information can be downloaded at: https://www.mdpi.com/article/10.3390/toxics13121052/s1, Supplementary Methods; Figure S1. The distribution of the sampling sites; Figure S2. The rarefaction curves of individual samples; Figure S3. Mechanisms of bacterial community assembly between pesticide-contaminated and pesticide-free soils using iCAMP model; Figure S4. Bacterial co-occurrence networks between pesticide-contaminated and pesticide-free soils at the OTU levels; Figure S5. Number of individuals and types in 3–8-node subgraphs in bacterial networks; Figure S6. The crash threshold values in bacterial networks under three attack types; Figure S7. Dynamics of relative concentrations of different 3-node subgraphs in bacterial networks under different attacks; Figure S8. Number of OTUs that significantly changed their abundance in response to abiotic factors of whole bacterial community; Figure S9. Functional prediction analysis based on Tax4Fun. Table S1. General description of sample information; Table S2. All connected 3-nodes and 4-nodes network subgraphs; Table S3. Resilience of subgraphs of bacterial network under different attacks; Table S4. Chemical properties and pesticide residues within greenhouse soil.
