# Supplementary material for: Pesticide Residues Reduce Bacterial Diversity but Enhance Stability via Network Motif Restructuring
Source: Toxics. 2025 Dec 4;13(12):1052. doi: 10.3390/toxics13121052 (PMC12737729; doi:10.3390/toxics13121052)
Supplement: Supplementary file 1 [file toxics-13-01052-s001.zip › toxics-4000990-supplementary.pdf]

SUPPLEMENTARY INFORMATION

Pesticide Residues Reduce Bacterial Diversity but Enhance Stability via Network Motif Restructuring

Chaonan Wang [1,2], Ruilin Wu [1], Xingyan Xue [3], Cunlu Li [1], Shengxing Long [1] and Fuliu Xu [1,*]

1 MOE Laboratory for Earth Surface Processes, College of Urban & Environmental Sciences, Peking University, Beijing 100871, China

2 State Key Laboratory of Advanced Environmental Technology, Department of Environmental Science and Engineering, University of Science and Technology of China, Hefei 230026, China

3 CCCC Water Transportation Consultants Co., Ltd., Beijing 100007, China

* Correspondence: xufl@urban.pku.edu.cn

This supporting information includes the following:

Supplementary Methods

Figure S1. The distribution of the sampling sites.

Figure S2. The rarefaction curves of individual samples.

Figure S3. Mechanisms of bacterial community assembly between pesticide-contaminated and pesticide-free soils using iCAMP model.

Figure S4. Bacterial co-occurrence networks between pesticide-contaminated and pesticide-free soils at the OTU levels.

Figure S5. Number of individuals and types in 3–8-node subgraphs in bacterial networks.

Figure S6. The crash threshold values in bacterial networks under three attack types.

Figure S7. Dynamics of relative concentrations of different 3-node subgraphs in bacterial networks under different attacks.

Figure S8. Number of OTUs that significantly changed their abundance in response to abiotic factors of whole bacterial community.

Figure S9. Functional prediction analysis based on Tax4Fun.

Table S1. General descriptions of sample information.

Table S2. All connected 3-nodes and 4-nodes network subgraphs.

Table S3. Resilience of subgraphs of bacterial network under different attacks.

Table S4. Chemical properties and pesticide residues within greenhouse soil.

Supplementary method

Soil pH was measured after creating a soil suspension, which consisted of a soil/water ratio of 1:2.5 (weight/g: volume/ml), with a pH meter according to the standard protocol (Sartorius). TOC was determined using TOC-5000A equipped with SSM-5000A solid sampler (Shimadzu, Japan) by subtraction. Firstly, total carbon (TC) was measured by burning at 900°C, and inorganic carbon (IC) was measured by adding 200 μL 50% phosphate acid and heating at 200°C. Then, TOC was calculated by TC minus IC.

Soil samples were properly mixed, and 5 g of sample was transferred into a centrifuge tube in three replicates and mixed with 5 mL pure water and 10 mL acetonitrile. The mixture was shaken for 3 min on a vertical shaker and treated with dispersive SPE magnesium sulfate ($MgSO_4$) Extraction Pouch ($MgSO_4$ 6 g, sodium acetate 1.5 g) to absorb any excess of water and shaken for 3 min. After 3 min, the content was vortexed and centrifuged at 5000 rpm for 5 min. The supernatant was filtered through 0.22 μM polytetrafluoroethylene (PTFE) filter papers before high-performance liquid chromatography (HPLC) analysis. Instrument calibration was performed using a series of standard solutions, yielding correlation coefficients (R^2) exceeding 0.99 for all targeted pesticides. The limits of detection (LOD) and quantification (LOQ) were calculated based on signal-to-noise ratios (S/N) of 3 and 10, respectively. The overall average recovery of pesticides from soil samples varied from approximately 89.32% to 96.15% in the final extracted solution. The relative standard deviations (RSDs) for pesticide extraction within each run of soil samples ranged from 2% to 5%, indicating that the variation between soil samples was relatively small and within control. Pesticides were determined

with HPLC (Waters I-CLASS, USA) using a C18 column (1.7 μm, 2.1 × 100 mm) at 40 °C. Acetonitrile (HPLC grade) and pure water 0.1% formic acid (HPLC grade) were used at a ratio of 70:30 and a flow rate of 0.4 mL/min.

The PCR amplification was performed as follows: initial denaturation at 95 °C for 3 min, followed by 27 cycles of denaturation at 95 °C for 30 s, annealing at 55 °C for 30 s, and extension at 72 °C for 45 s, with a final extension at 72 °C for 10 min. PCR reactions were performed in triplicate in a 20 μL mixture containing 4 μL of 5× FastPfu Buffer, 2 μL of 2.5 mM dNTPs, 0.8 μL of each primer (5 μM), 0.4 μL of FastPfu Polymerase (TransGen, Beijing, China), and 10 ng of template DNA.

Supplementary Figures

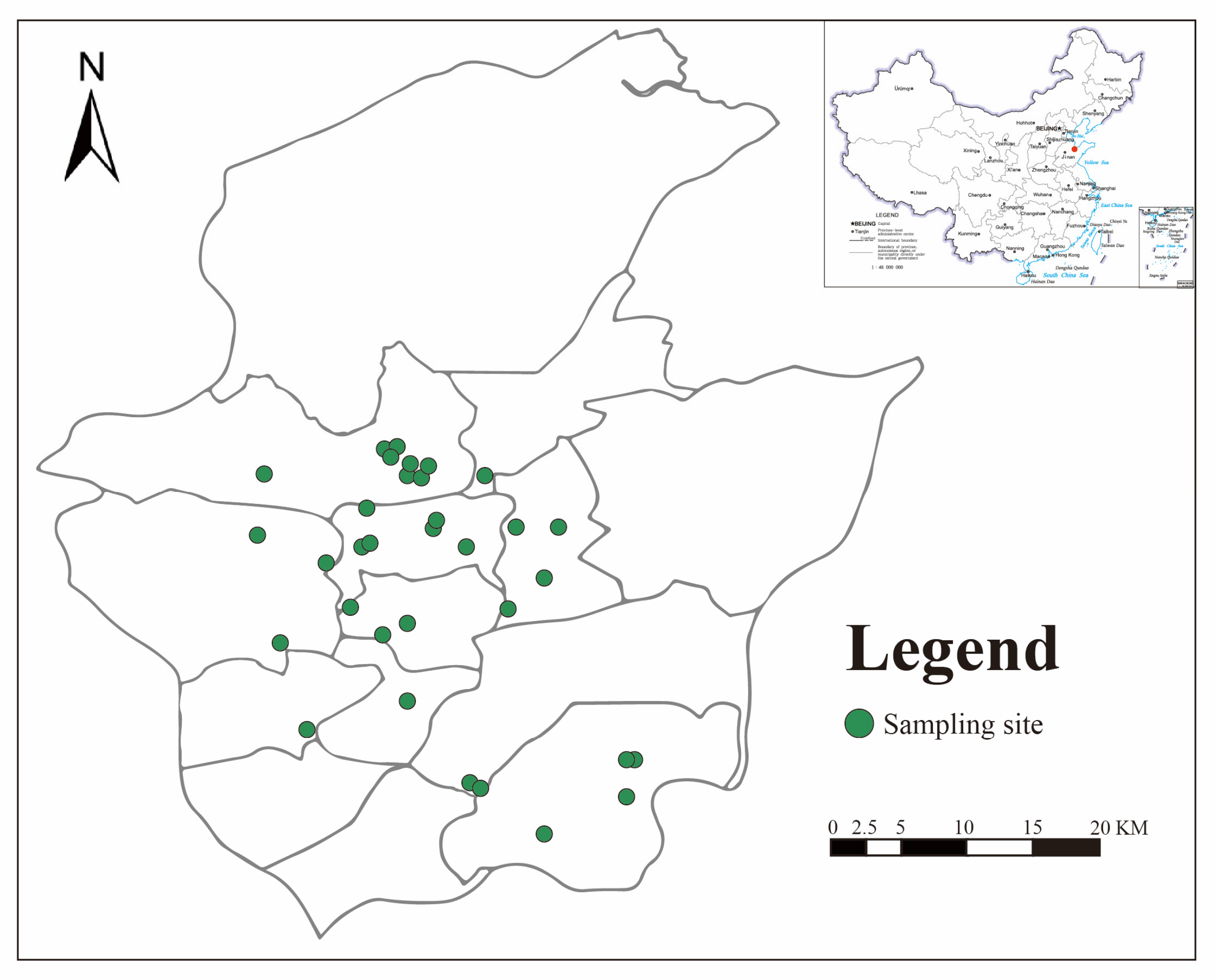

Figure S1. The distribution of the sampling sites.

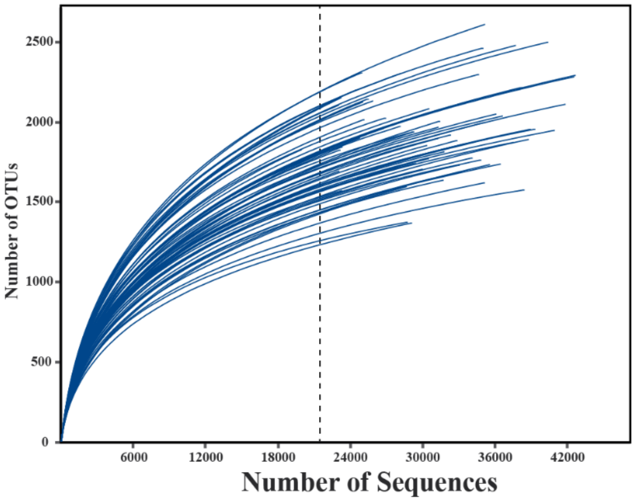

Figure S2. The rarefaction curves of individual samples. Rarefaction curves were assembled to show the number of OTUs, defined at 97% sequence similarity cut-off in mothur, relative to the number of total sequences. The dashed vertical line indicates the number of sequences (21982 reads per sample) subsampled from each sample to calculate alpha diversity estimates.

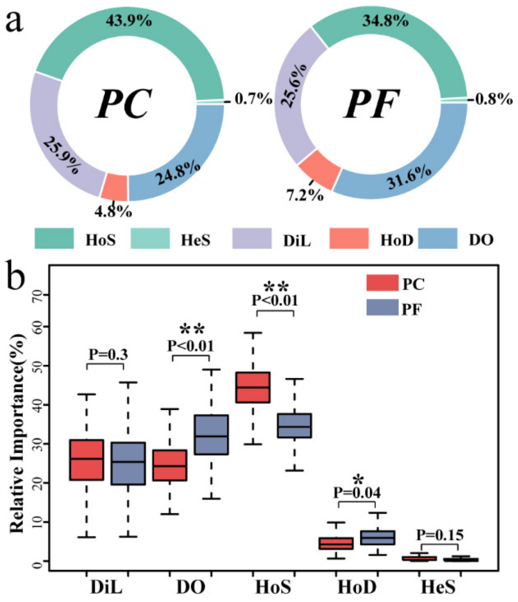

Figure S3. Mechanisms of bacterial community assembly between pesticide-contaminated and pesticide-free soils using iCAMP model. HoS: homogeneous selection; HeS: heterogeneous selection; DiL: dispersal limitation; HoD: homogenizing dispersal; DO: drift and others.

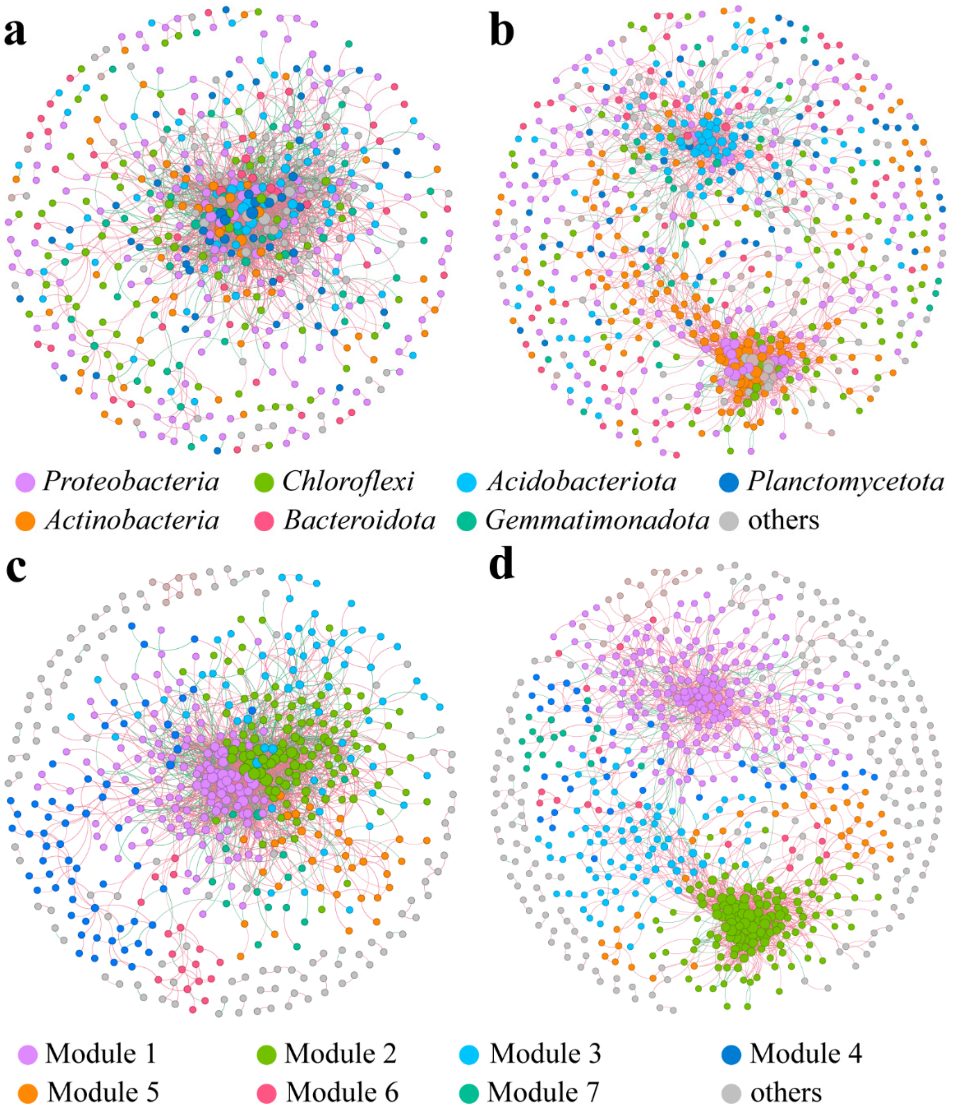

Figure S4. Bacterial co-occurrence networks between pesticide-contaminated and pesticide-free soils at the OTU levels. The network of pesticide-contaminated soils (**a**) and pesticide-free soils (**b**), of which nodes were colored by phylum assignment. The network of pesticide-contaminated soils (**c**) and pesticide-free soils (**d**) of which nodes were colored by modules. Red lines represent positive correlation while green lines represent negative correlation in networks.

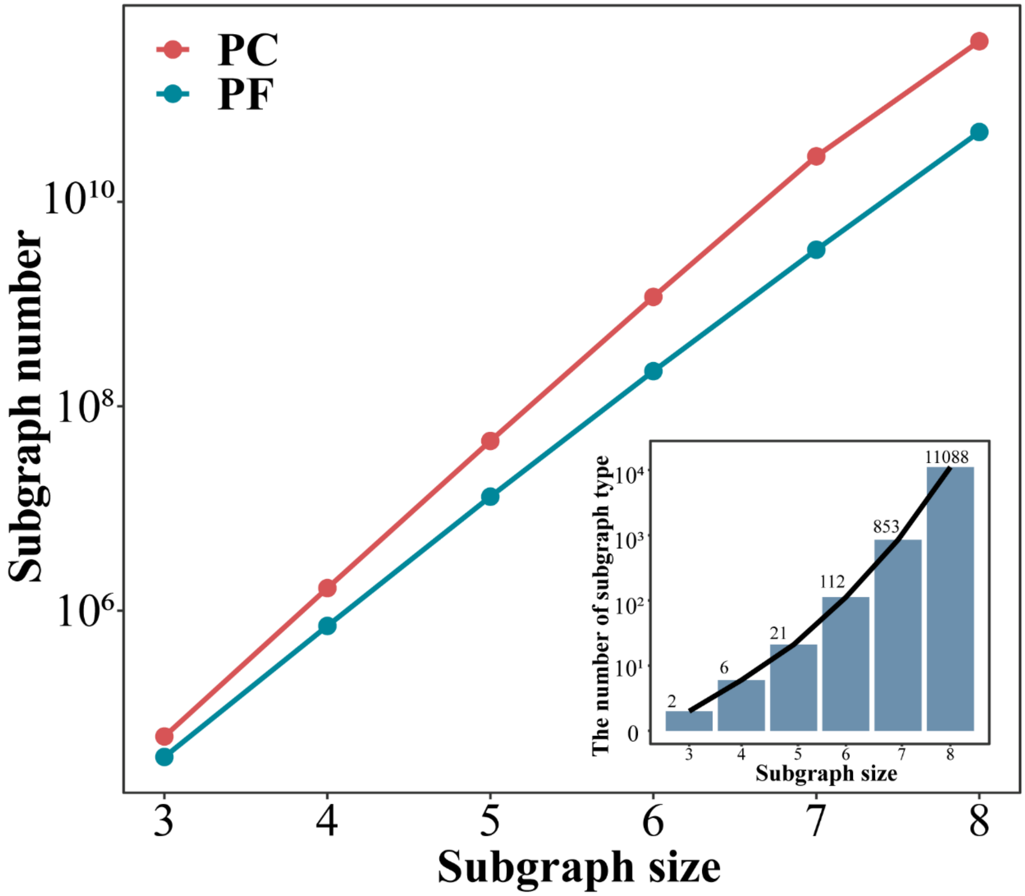

Figure S5. Number of individuals and types in 3–8-node subgraphs in bacterial networks.

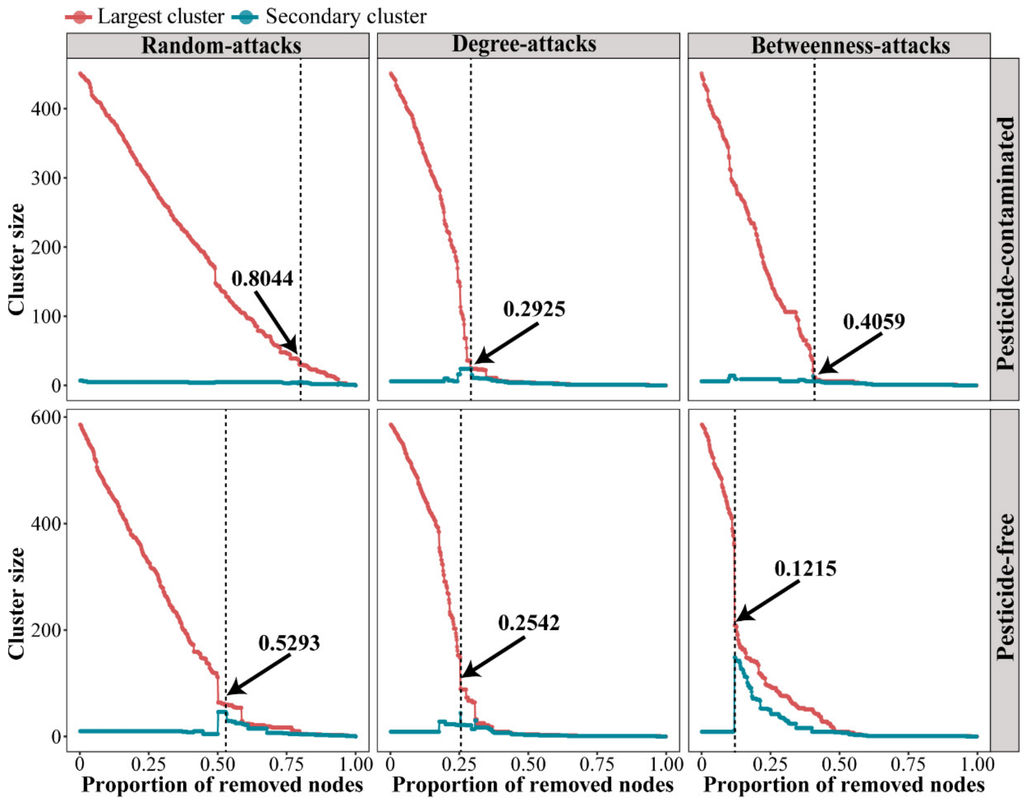

Figure S6. The crash threshold values in bacterial networks under three attack types. The bold numbers represent threshold values.

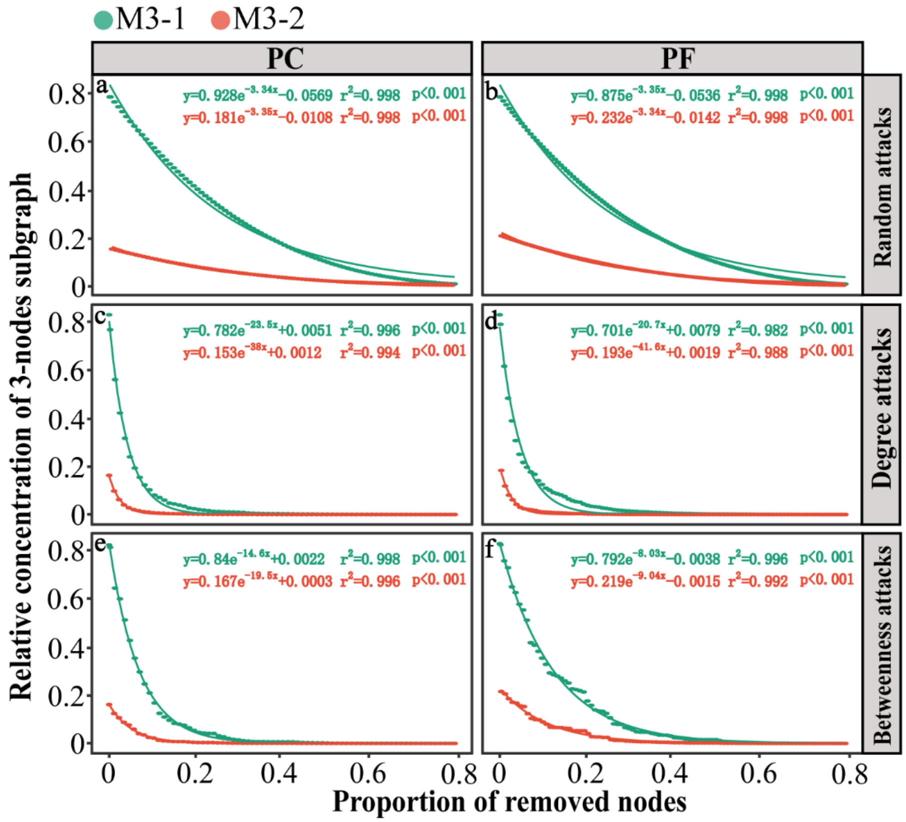

Figure S7. Dynamics of relative concentrations of different 3-node subgraphs in bacterial networks under different attacks. (**a**) Network of greenhouse under random-based attacks; (**b**) network of open field under random-based attacks; (**c**) network of greenhouse under degree-based attacks; (**d**) network of open field under degree-based attacks; (**e**) network of greenhouse under betweenness-based attacks; (**f**): network of open field under betweenness-based attacks. Larger shifts upon the same proportion indicate that there is less stability within subgraph types.

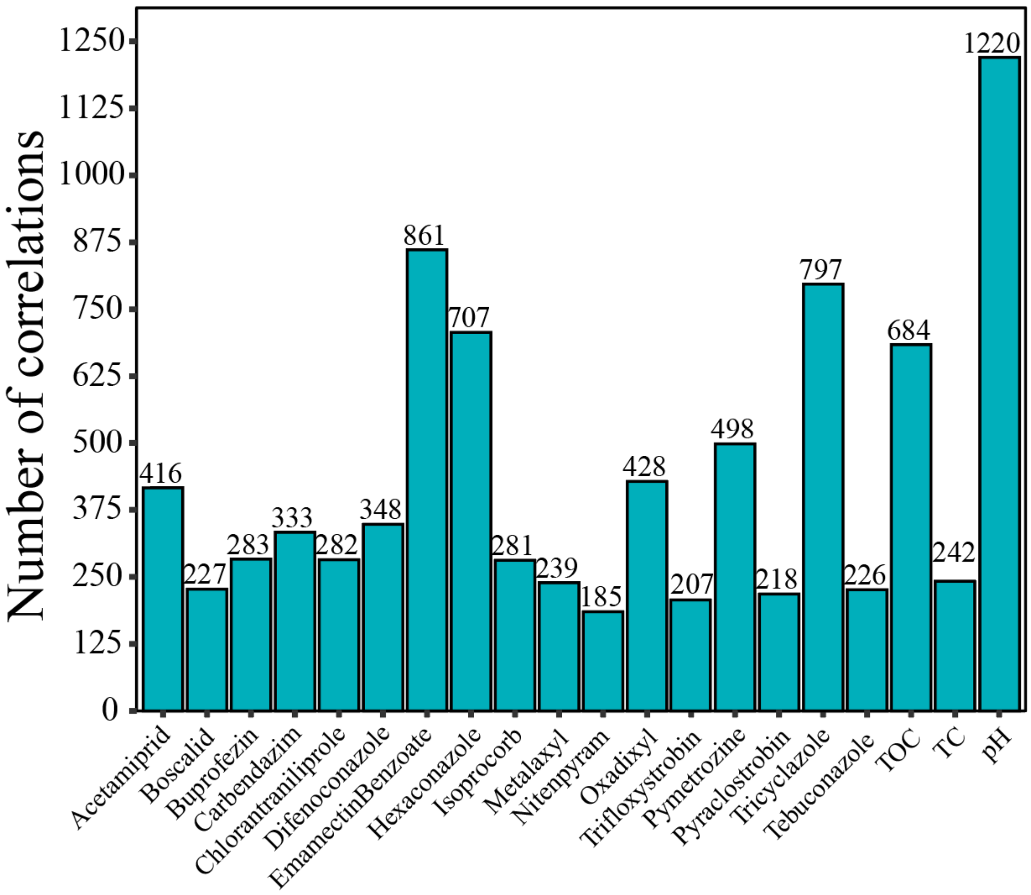

Figure S8. Number of OTUs that significantly changed their abundance in response to abiotic factors of whole bacterial community. The number at the top of the bar represents the number of OTUs significantly related to corresponding environmental variable.

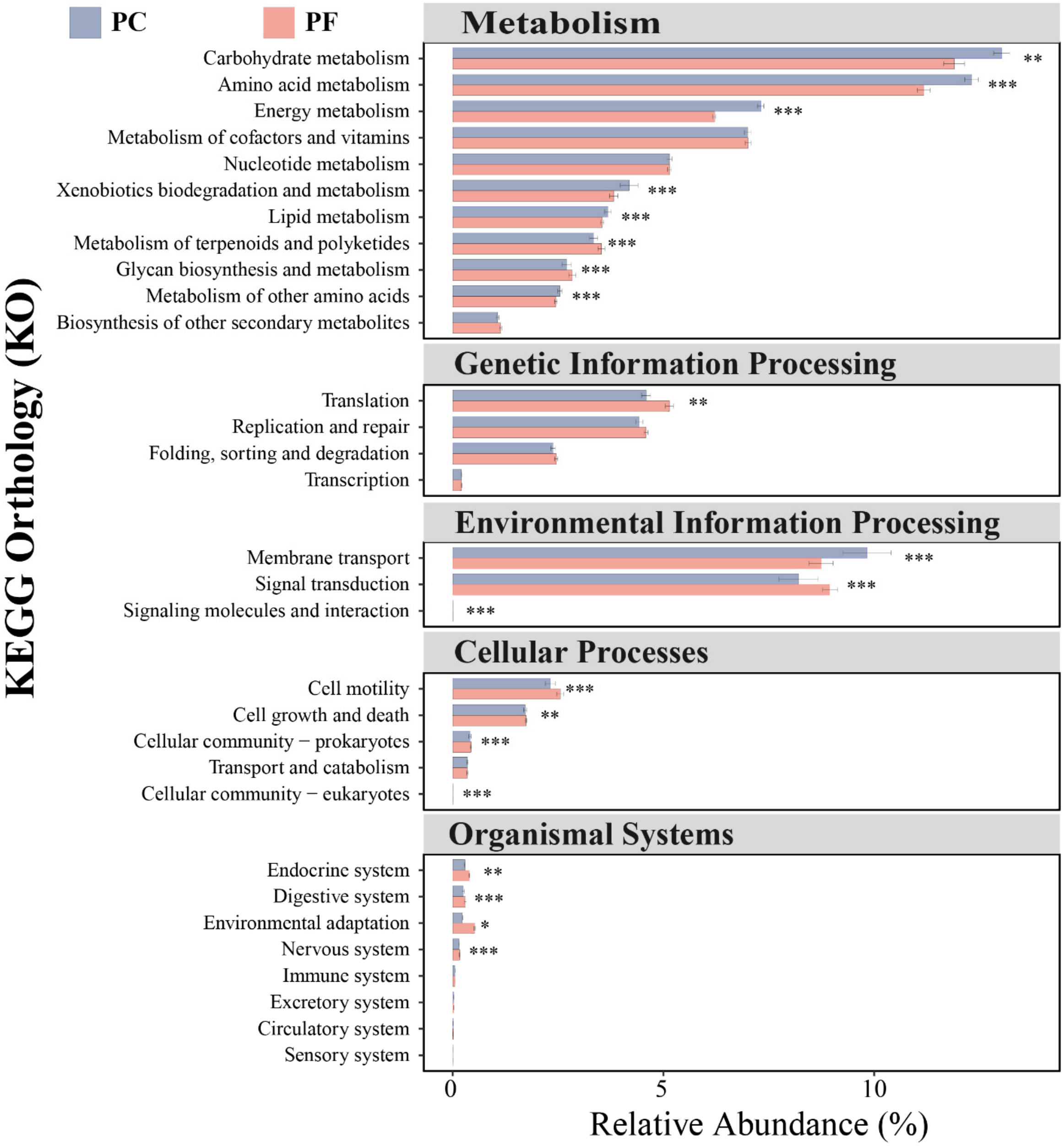

Figure S9. Functional prediction analysis based on Tax4Fun. *: $p < 0.05$, **: $p < 0.01$, ***: $p < 0.001$.

Supplementary Tables

Table S1. General descriptions of sample information.

Sites	Planting age	Crop type	Longitude	Latitude
1	16	tomato	118.74533443E	36.97092751N
10	17	tomato	118.92085919E	36.81017736N
11	22	tomato	118.92867557E	36.80841045N
12	10	tomato	118.90030777E	36.75209886N
13	18	tomato	118.89896717E	36.74844489N
14	15	tomato	118.87828264E	36.77238034N
15	9	tomato	118.87524982E	36.78426022N
16	3	tomato	118.88117981E	36.78520203N
17	13	tomato	118.68866238E	36.97248361N
18	14	tomato	118.68664493E	36.9723329N
19	9	tomato	118.71565159E	36.9960907N
2	2	tomato	118.72817106E	36.96532186N
20	12	tomato	118.71681213E	36.99860764N
21	10	tomato	118.75592379E	36.99367486N
22	2	tomato	118.79515680E	37.02167285N
23	27	tomato	118.79739879E	36.97570465N
24	9	cucumber	118.79649599E	37.03109958N
25	21	tomato	118.78545793E	37.02297578N
26	2	cucumber	118.77323083E	37.03770773N
27	13	cucumber	118.77312957E	37.03787800N
28	12	cucumber	118.77504090E	37.03960254N
29	2	tomato	118.81832195E	36.948828N
3	8	tomato	118.72904571E	37.00849893N
30	12	tomato	118.80786099E	36.92890245N
31	5	tomato	118.89494545E	36.85631186N
32	4	tomato	118.89301451E	36.76981095N
33	20	cucumber	118.82201527E	36.81469913N
34	15	cucumber	118.82638156E	36.81256968N
5	21	tomato	118.74614504E	36.93756460N

6	11	tomato	118.75381733E	36.97538128N
7	8	tomato	118.75666458E	36.99956303N
Sites	Planting age	Crop type	Longitude	Latitude
8	3	tomato	118.93240820E	36.83495389N
9	3	cucumber	118.93000829E	36.83463668N

The open field samples at sites 27 and 28 are the same and the open field samples at sites 29 and 30 are the same due to the close proximity of two points.

Table S2. All connected 3-nodes and 4-nodes network subgraphs.

ID	Subgraph Shape	Structural Description	Potential Ecological Interpretation
M3-1	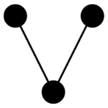	A core species interacts with each of the other two non-interacting species.	Open triplet (Anti-motif); represents simple mediated interactions with low local clustering and low redundancy.
M3-2	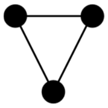	Three species interact in pairs.	Closed triplet (Motif); represents transitive connectivity and high local clustering, often indicating a stable cooperative functional unit or "guild".
M4-1	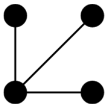	A core species interacts with each of the other three non-interacting species.	Star/Hub topology; suggests a central "keystone" species regulating multiple peripheral species; sensitive to the removal of the center.
M4-2	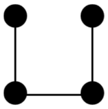	Two intercropping species interact separately with two other non-intercropping species.	Chain-like topology; represents linear information/energy transfer with low efficiency and high vulnerability to interruption.
M4-3	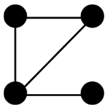	Three species interact with each other, and the fourth species interacts with one of them.	Intermediate clustering; a transitional state between a closed triplet and a fully connected clique.
M4-4	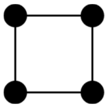	Among the four species, each interacts with only two species, respectively.	Cycle topology; represents a feedback loop involving four species, with moderate redundancy.
M4-5	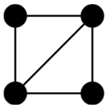	Four species interact in pairs, except that certain two species do not interact.	High clustering; represents a robust module reinforced by cross-links, enhancing local stability.
M4-6	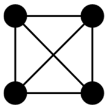	Four species interact in pairs	Clique (Motif); represents the maximally connected 4-node subgraph, indicating the highest level of redundancy, cooperation, and resistance to node loss.

Table S3. Resilience of subgraphs of bacterial network under different attacks.

ID	Random-based attacks		Degree-based attacks		Betweenness-based attacks	
	PC	PF	PC	PF	PC	PF
M3-1	0.2132	0.1885	0.0347	0.0357	0.0583	0.061
M3-2	0.0417	0.0501	0.0044	0.0051	0.0087	0.016
M4-1	0.0473	0.0376	0.0056	0.005	0.0109	0.0135
M4-2	0.0797	0.0686	0.0119	0.0112	0.0213	0.0243
M4-3	0.0595	0.0631	0.0057	0.006	0.0121	0.0225
M4-4	0.0015	0.0016	0.0003	0.0002	0.0004	0.0006
M4-5	0.0103	0.0193	0.0011	0.0014	0.0025	0.0069
M4-6	0.0034	0.0068	0.0002	0.0004	0.0001	0.0023

Table S4. Chemical properties and pesticide residues within greenhouse soil.

Sites	pH	TC	TOC	EmB	Chl	Tri	Dif	Pyr	Bos	Hex	Teb	Bup	Meta	Oxa	Nit	Ace	Pym	Iso	Carb	Tri
1	7.215	24.92	24.053	1.71	9.9	0	21.38	25.13	94.88	0.44	26.93	0.44	0.83	2.08	7.6	0.43	0.05	0.64	7.98	5.43
2	7.605	18.01	9.226	0.19	0.13	0.01	17.12	5.34	45.68	0.95	7.56	0.08	5.79	0.09	67.23	1.06	4.1	0.31	17.9	1.25
3	7.6	21.22	11.911	0.25	3.54	0	2.84	0.11	11.27	0.1	4.85	0.52	0.24	0.02	9.46	0.16	0.05	0.12	0.99	0.56
5	7.3	13.38	12.815	0.18	33	0.52	16.44	17.03	218.8	1.82	1.22	1.75	6.05	0.31	222.6	3.09	0.01	0.78	5.37	1.12
6	7.27	12.64	11.754	0.19	2.41	0.01	2.71	14.57	79.48	0.43	26.93	0.08	0.41	0.3	46.33	0.12	1.18	0.27	3.84	0.44
7	7.685	14.52	10.736	0.8	18.81	1.18	11.62	9.03	77.73	0.05	8.41	0.01	2.98	0.07	116.7	1.43	0	1.48	6.14	0.79
8	7.54	11.48	8.451	0.15	2.58	0.01	0.82	2.34	12.54	0.11	0.32	0.08	0.71	0	0.97	0.01	0	0.12	7.77	5.7
9	7.25	14.58	11.834	16.1	0.74	0	54.1	6.07	4.81	0.14	11.76	0.04	1.38	0.04	390.9	0.14	0.02	0.37	10.53	0.89
10	7.37	27.33	23.852	1.76	41.78	0	106	0.08	60.98	0.21	2.58	1.29	3.12	1.45	344.1	1.9	0	0.96	190.4	0.54
11	7.385	21.93	13.515	5.23	10.53	0.02	12.62	0.43	64.08	0.37	14.66	0.88	2.42	0.87	62.13	0.41	2.72	0.62	13.23	15.04
12	7.55	15.41	13.618	0.25	15.79	10.36	23.1	17.3	54.65	0.09	24.28	0.28	0.24	0.06	19.28	1.22	7.52	1.3	4.19	2.57
13	7.185	12.51	11.953	0.36	7.45	0.64	9.44	0.37	56.2	0.01	1.84	0.22	0.55	0.1	66.1	3.14	0.62	1.68	4.93	0.8
14	7.195	19.26	14.784	3.59	4.16	0.08	5.98	10.6	27.8	1.78	6.89	1	0.14	2.63	8.18	0.38	1.65	0.25	0.77	0.4
15	7.32	19.69	15.707	0.72	14.77	0.4	13.92	6.54	132.4	0.67	22.9	0.42	0.47	0.19	15.42	0.42	0.06	0.2	2.11	2.3
16	7.64	10.63	8.877	0.01	17.49	0	0.2	0.06	1.11	0.02	0.42	0.02	0.15	0.02	0	0.05	0	0.03	0.96	0.01
17	7.39	28.87	20.373	0.63	23.53	0.15	11.45	17.61	28.6	0.18	9.8	2.23	0.53	0.02	37.03	0.64	0.7	0.89	1.34	0.16
18	7.76	22.44	10.27	0.62	0.15	0.06	17.49	21.55	11.08	0.29	1.06	0.77	2.14	0.08	16.63	0.94	0.03	1.06	21.7	0.13
19	7.565	20.00	7.55	0.08	0.39	0.02	5.78	5.58	122.9	0.19	0.31	4.64	0.15	0.02	71.43	0.49	0.01	0.36	2.17	0.17
20	7.815	18.56	11.328	0.19	0.99	0.52	14.53	1.43	14.97	0.13	13.95	0.18	2.5	0.03	43.8	0.26	0.33	0.37	5.16	0.04
21	7.45	18.16	14.452	0.09	19.57	2.7	22.68	2.13	22.08	0.12	18.55	0.23	2.97	0.24	52.9	1.13	0.02	0.05	5.4	0.76

Site	pH	TC	TOC	EmB	Chl	Tri	Dif	Pyr	Bos	Hex	Teb	Bup	Meta	Oxa	Nit	Ace	Pym	Iso	Carb	Tri
22	7.6	18.41	14.665	0.23	0.29	0.86	10.14	5.26	90.38	0.32	11.99	0.06	0.18	0	11.44	0.1	0	2.16	2.52	2.95
23	7.32	11.91	10.415	0.3	3.11	0.04	5.89	2.3	19.1	0.01	1.21	0.4	2.53	0.28	9.18	0.19	0.01	0.63	40.62	0.13
24	7.07	26.81	23.569	1.59	0.55	0	22.93	0.58	0.71	1.06	1.99	0.62	0.38	0	30.8	0.38	0.02	0.27	10.57	1.75
25	7.23	16.04	15.051	2.87	3.98	0.58	6.78	1.28	112.3	0.23	13.58	0.44	2.97	0.08	18.17	0.58	0.62	1	17.31	0.86
26	7.26	13.46	12.316	9.72	1.51	0.02	18.27	0.15	0.75	0.2	0.64	0.52	0.88	0.01	54.53	0.04	0.06	0.25	20.47	2.52
27	6.705	15.23	13.876	11.6	1.35	0.01	20.67	2.08	0.29	0.24	3.64	83.86	0.82	0.01	116.0	0.21	0.1	0.49	7.67	4.46
28	6.545	24.6	22.241	2.18	6.74	1.13	35.23	3.79	12.14	0.7	16.16	0.07	11.1	0	3.4	0.17	0.03	0.34	103.8	3.44
29	6.915	14.11	10.613	0.63	1.18	0.82	2.96	0.99	0.1	0.17	5.05	0.05	1.25	0.01	7.18	0.04	3.96	0.22	3.5	0.37
30	6.98	15.23	13.209	7.31	13.41	0.29	2.78	0.2	1.89	1.19	5.65	0.65	0.21	0.05	31	0.21	0.99	1.65	6.55	0.56
31	7.49	18.63	14.722	0.89	6.43	0.3	25.23	1.07	43.03	1.79	4.14	0.05	1.57	0.63	4.47	0.39	0.24	0.12	4.18	0.76
32	7.385	16.38	14.628	0.11	1.85	0.41	3.96	1.51	18.45	0.05	0.46	0	0.83	4.35	7.97	1.61	0.01	0.11	0.01	0.21
33	7.505	19.31	18.263	1.61	9.71	0.13	26.68	10.46	55.1	0.38	8.1	0.32	1.31	0.88	143.1	2.29	0.06	0.12	19.98	1.65
34	7.45	24.91	21.346	1.76	1.56	0.05	38.6	2.8	76.65	0.31	1.69	0.15	1.31	0.35	59.1	0.51	0.06	0.08	32.83	1

TC: Total carbon (g/kg); TOC: (g/kg); Total organic carbon; EmB: Emamectin Benzoate; Chl: Chlorantraniliprole; Tri: Trifloxystrobin; Dif: Difenoconazole; Pyr: Pyraclostrobin; Bos: Boscalid; Hex: Hexaconazole; Teb: Tebuconazole; Bup: Buprofezin; Meta: Metalaxyl; Oxa: Oxadixyl; Nit: Nitenpyram; Ace: Acetamiprid; Pym: Pymetrozine; Iso: Isoprocorb; Car: Carbendazim; Tri: Tricyclazole. The unit of pesticide residues are μg/kg.
